# Editorial: Current innovations in GI-oncology: Where do we stand?

**DOI:** 10.3389/fonc.2022.1048082

**Published:** 2022-10-27

**Authors:** Holger Rumpold, Dominik Wolf

**Affiliations:** ^1^ Gastrointestinal Cancer Center, Ordensklinikum Linz, Linz, Austria; ^2^ Medical Faculty, Johannes Kepler University, Linz, Austria; ^3^ Internal Medicine 5, Department of Hematology and Oncology, Comprehensive Cancer Center Innsbruck (CCCI), Medical University Innsbruck (MUI), Innsbruck, Austria

**Keywords:** gastrointestinal cancer, multimodal treatment, molecular markers, microbiome, rare cancers

Estimations on incidence and mortality due to cancer worldwide show 4.8 million new cancer cases and 3.4 million deaths related to cancer worldwide in 2018. Cancers of the gastrointestinal (GI) tract represents 26% of this global cancer incidence and 35% of deaths caused by cancer worldwide. These numbers are predicted to increase to 58% (incidence) and 73% (mortality) until 2040, which is mainly due to the expected changes in the age composition and growth of the world population (1). Over the recent centuries, the options for treating GI-cancers have been increasing significantly. However, these are not only based on highly sophisticated novel treatments known under terms like “precision oncology” or “immunoncology” but also on clinically based inventions like multimodal treatment approaches including, for example, metastasectomy on the one hand and organ sparing strategies on the other hand in colorectal cancer. A few cutting edge papers covering clinical as well as basic scientific problems have been chosen for this Research Topics to reflect the broad field of current innovations in GI-oncology. The common topic of the contributions to this series is the still ongoing debate about risk-adapted treatment strategies including modern biomarkers as well as clinical features.

Such, modern treatments including organ sparing strategies for patients at low-risk for recurrence represent one of the most intriguing developments over the last years. Whereas response triggered decisions for watch and see strategies in high-risk rectal cancer have already entered clinical routine, the same question is still a matter of debate in early stage, low risk colorectal cancer. Organ sparing is of emphasized interest in an elderly population, which has been retrospectively investigated in one of the papers in this series (Ye et al.). The authors were able to show in a large number of patients with T1 colorectal cancer documented in the Surveillance, Epidemiology, and End Results database between 2004 and 2015, that in patients > 65 years with adenocarcinoma, a tumor diameter < 3 cm and negative CEA showed lymph node involvement in 4.6% only. The number of lymph nodes removed (more or less than 12) were not associated with cancer specific survival in this patient cohort. This large analysis shows that organ sparing treatment strategies should be investigated more intensively in the future. It further fits and supports several other authors, which focused on sentinel lymph node procedures in early colorectal cancer (2–4). Taken this together, organ sparing procedures are of high clinical importance – not only on a scientific level as it also goes along with higher quality of life for patients.

The discussion of intensifying treatment or not is well developed for colorectal cancer but much less for other entities like gastric cancer. In this series, Sun et al. publish a systematic literature review covering the question, if multimodal treatment prolongs survival in patients with gastric cancer and liver metastasis. They included 23 studies and more than 5000 patients for their review. Taken together, multimodal treatment including systemic treatment, surgery as well as ablative treatments in combination lead to a remarkable prolongation of survival if compared to less intensive treatment. Together with others, the review contributes to an ongoing discussion and underlines that multimodal treatment approaches should be considered in a special patient population (5). Key for success is a qualified multidisciplinary team for decision making and treating these patients in a coordinated and experienced manner to provide a balanced risk-benefit ratio.

Next to the more common entities in the GI-tract, Low-Grade Appendiceal Mucinous Neoplasms (LAMN) represents a very rare disease. Additionally, the WHO and other organization proposed an adaption of these tumors as biological differences are of high prognostic relevance. In this series, Lu et al. contribute a case series of 22 patients with LAMN confined to the appendix. These are often associated with diverticula and could be misdiagnosed as serrated lesions. The correct interpretation of the findings are of importance as prognosis and treatment differ. Namely, in the cases investigated, the authors were not able to detect a short-term benefit of prophylacted extended resection of hyperthermic intraperitoneal chemotherapy, which are hallmarks in the treatment of these tumors in other settings.

Intratumor heterogeneity (ITH) is among the biggest hurdles of successful treatment and also limits the power of relatively small biopsies taken to detect prognostic or even therapeutically relevant targets. This relates to the subclonal evolution of the diseases, which has first been highlighted from the genetic point of view by Gerlinger et al. (6). Qin et al now compared a systematic sampling method (SSM) with a general sampling method (GSM) to obtain tumor tissues, tumor and normal squamous epithelium biopsies from 81 esophageal cancers and focused on the intratumor-heterogeneity (ITH) leading to insufficient cancer diagnosis and positive rates of P16 methylation. The positive rates of P16 methylation was substantially higher in SSM vs. GSM (94 vs 63%). The overall concordance of pathological diagnosis and P16 methylation between tumor biopsy and the corresponding tumor tissue was 75.0% and 62.5%, respectively. This data highlights the limitations of biopsies taken from suspected tumors and that the SSM-technology, even though being more laborious, provides a higher diagnostic accuracy. The ITH may at least in part explain these data, leading to the discordance between the techniques. However, more studies with larger sample sizes, companion biomarkers, and high resolution methylation testings are required to define novel standardized techniques for a more accurate diagnostic work-up in esophageal cancer.

ITH may also be relevant for changes affecting somatic alterations of the DNA-damage repair machinery, an issue summarized by Zimmer et al. This does certainly not hold true for germline mutations of DNA damage repair (DDR) genes, such as the “breast cancer gene 1” (BRCA1) and BRCA2, that have originally been identified as major susceptibility genes in breast and ovarian cancers and that are present in all tumor cells. However, with the establishment and also the approval of more cost-effective next generation gene sequencing (NGS) methods, not only germline but also somatic BRCA mutations have been identified in a wide variety of cancer types. This is of utmost importance, as the approval of poly (ADP)-ribose polymerase inhibitors (PARPi) for BRCA-mutated cancers (e.g., ovarian and pancreatic cancer) (7, 8), analysis of BRCA mutations has important therapeutic implications by improving outcome. However, also other genes involved in the DDR pathway, such as ATR, ATM, PALB, WRN and CHK1, have emerged as potential new treatment targets, and inhibitors of these proteins are currently under clinical investigation in early clinical phase trials. As the frequency of germline and somatic BRCA- and other HRR gene-mut GI cancers varies extensively between different sites within the GI-tract, the clinical implications of such findings are determined in a variety of currently and future running clinical trials. Moreover, investigating the BRCAness phenotype (in addition to solely defining BRCA1 or BRCA2 mutations), is gaining more and more attention as it may offer novel promising combination therapy options.

Finally, a large proportion of GI-cancer patients suffer from metastatic disease, which requires systemic therapies, mainly chemotherapy. Various host-related factors contribute to the efficacy of systemic therapies (i.e. co-morbidity burden, age etc.). Another detrimental factors is the gastrointestinal (GI)-microbiom, that is highly relevant for a variety of pathophysiological conditions, including also the pathogenesis of GI-cancer (9). The high relevance of microbes for cancer development and growth has recently been acknowledged by integration of ‘polymorphic microbiomes’ to the most recent update of the “Hallmarks of Cancer” concept (10). This is in part based on the ground-breaking observations that the host microbial composition is linked to the efficacy of ICI therapies (11, 12), thereby also offering therapeutic potential (11, 13). Recently, Oh et al. - by providing systematic review in Frontiers of Oncol - highlighted the impact of the gut microbiome for chemotherapy in GI-oncology. Several studies support the concept that the gut microbiome is linked to both CTX, chemotherapy efficacy and toxicity. Ten studies prospectively assessed the impact of CTX on GI-microbiome composition during treatment and highlighted that chemotherapy induces substantial dysbiosis, which is also related to the appearance of adverse events. Large systematic analyses of the GI-microbiome prior and during therapy with a detailed correlation to inflammatory and immunological biomarkers as well as adverse event and clinical outcome are required to better define how future interventional studies targeting the GI-microbiota should be planned.

## Author contributions

Both authors contributed equally to the editorial (conceptualization, writing, proofreading). All authors contributed to the article and approved the submitted version.

## Conflict of interest

The authors declare that the research was conducted in the absence of any commercial or financial relationships that could be construed as a potential conflict of interest.

## Publisher’s note

All claims expressed in this article are solely those of the authors and do not necessarily represent those of their affiliated organizations, or those of the publisher, the editors and the reviewers. Any product that may be evaluated in this article, or claim that may be made by its manufacturer, is not guaranteed or endorsed by the publisher.
